# DNA methylation dictates histone modifications in developing male germ cells in the mouse

**DOI:** 10.1093/nar/gkaf1240

**Published:** 2025-11-20

**Authors:** Hirotaka Sugimoto, Masaki Kawase, Kenji Ichiyanagi

**Affiliations:** Laboratory of Genome and Epigenome Dynamics, Department of Animal Sciences, Graduate School of Bioagricultural Sciences, Nagoya University, Furo-cho, Chikusa-ku, Nagoya 464-8601, Japan

## Abstract

In the male germline of mammals, retrotransposon expression is restricted by DNA methylation, trimethylation of histone H3 at lysine 9 (H3K9me3), and PIWI-interacting small RNAs (piRNAs). To elucidate their relative importance in regulating retrotransposons during germ cell development and the relationships between these mechanisms, we performed mRNA sequencing, DNA methylation, and histone methylation analyses using mouse spermatogonia from *Dnmt3l* and *Pld6* mutants deficient in *de novo* DNA methylation and piRNA production, respectively. The results revealed that loss of DNA methylation resulted in decreased H3K9me3 in young L1 subfamilies and increased H3K4me3 in many retrotransposons, suggesting a pivotal role of DNA methylation in maintaining epigenomic integrity in spermatogonia and later stages of spermatogenesis. The transcriptional up-regulation of retrotransposons by a loss of DNA methylation was more evident during meiosis (spermatocytes) than before meiosis (spermatogonia). These results are aligned with a global reduction of H3K9me3 at retrotransposons in spermatocytes. The piRNA system also regulated H3K9me3 and H3K4me3 at retrotransposons in spermatogonia, probably through the regulation of DNA methylation, since the loss of DNA methylation resulted in decreased H3K9me3 and increased H3K4me3 at the same retrotransposon loci even in the presence of piRNAs.

## Graphical Abstract

**Graphical Abstract:**
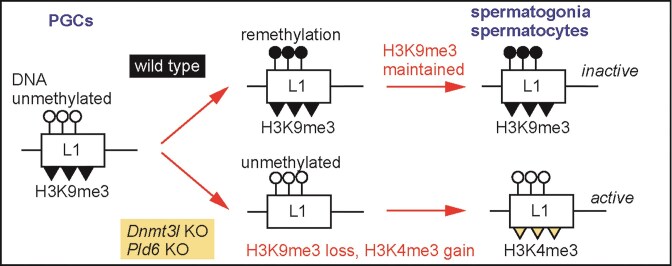

## Introduction

Retrotransposons, including long interspersed elements (LINEs), short interspersed elements (SINEs), and long terminal repeat (LTR) elements, are a group of transposable elements, which constitute 30–50% of the genome in mammals [1]. These retrotransposons can insert the sequences of their original copies into other genomic sites; therefore, their expression is tightly regulated by epigenetic mechanisms, including DNA methylation, trimethylation of histone H3 at lysine 9 (H3K9me3), and PIWI-interacting small RNAs (piRNAs). In male mouse germ cells, DNA methylation is erased genome-wide in primordial germ cells (PGCs) by embryonic day 13.5 (E13.5), and massive *de novo* methylation occurs in prospermatogonia from E14.5 to birth (postnatal day 0, P0) [2]. During this period, piRNAs are actively produced and enriched with sequences derived from evolutionarily young retrotransposons, whereas piRNAs produced in later developmental stages are enriched with unique sequences [3]. Prospermatogonial piRNAs bind to the PIWIL4 (also called MIWI2) protein to target the LINE promoter region for *de novo* DNA methylation and H3K9me3 [4, 5]. Moreover, piRNAs facilitate the cleavage of retrotransposon RNAs via a post-transcriptional silencing mechanism [6, 7]. DNA methylation [8] and H3K9me3 [9, 10] play important roles in transcriptional silencing of retrotransposons in male germ cells. However, the importance of DNA methylation is not evident in prospermatogonia but is evident in meiotic spermatocytes [7].

The roles of piRNAs in DNA methylation and H3K9me3 have been extensively studied; however, the interactions between DNA methylation and H3K9me3 are still unclear. H3K4me2/3 has been reported to regulate *de novo* DNA methylation in prospermatogonia [11, 12], while it remains unknown whether DNA methylation affects H3K4me2/3.

To address the above questions and elucidate the developmental changes in the importance of individual regulatory mechanisms, we studied retrotransposon expression in postnatal day 7 (P7) spermatogonia, which are between the prospermatogonial and spermatocyte stages, in the wild type as well as *Dnmt3l* knockout (KO) and *Pld6* KO mutants deficient for *de novo* DNA methylation and piRNA biogenesis, respectively. We also analyzed H3K9me3 and H3K4me3 at retrotransposons in these cells, which revealed that the loss of DNA methylation resulted in a decrease in H3K9me3 at young LINE copies and an increase in H3K4me3 at many retrotransposons.

## Materials and methods

### Mice

The generation of *Pld6* KO and *Dnmt3l* KO mice has been described previously [13, 14]. The mutant lines were backcrossed to C57BL/6J for >12 generations. Animal experiments were conducted in accordance with the Japanese Act on Welfare and Management of Animals, Guidelines for Proper Conduct of Animal Experiments (published by the Science Council of Japan), Fundamental Guidelines for Proper Conduct of Animal Experiments and Related Activities in Academic Research Institutions (published by the Ministry of Education, Culture, Sports, Science, and Technology, Japan), and the Regulation for Animal Experiments at Nagoya University. All the protocols were approved by the Animal Experiments Committee of Nagoya University.

### Purification of germ cells

To purify spermatogonia at P7, the testicular cells were labeled with an antibody (sc-53532, Santa Cruz Biotechnology) against EpCAM (CD326), a surface marker of undifferentiated spermatogonia [15], and Alexa Fluor 488-labeled anti-rat IgG antibody (ab150165, Abcam), as described previously [16]. The EpCAM-positive cells were sorted using an SH800 cell sorter (Sony, Tokyo, Japan) (Supplementary Fig. S1A). DNA methylation analysis for the *Lit1* imprinting differentially methylated region confirmed high purity (∼97%) of germ cells (Supplementary Fig. S1B).

To purify the leptotene and zygotene (L/Z) stages of spermatocytes, the testicular cells at P21 were stained with Hoechst 33 342, and L/Z spermatocytes were collected by fluorescence-activated cell sorting (FACS) using SH800 based on blue and red fluorescence as described previously [7] (Supplementary Fig. S1C). Immunofluorescent analysis using anti-Sycp3 and anti-γH2A.X antibodies confirmed that the majority of the sorted cells were spermatocytes of L or Z stage (Supplementary Fig. S1D).

### mRNA-seq library preparation and analysis

RNA was extracted using Isogen (Toyobo) and a DirectoZol RNA prep kit (ZymoResearch) from the spermatogonia of three, two, and three mice for wild-type, heterozygous, and homozygous KO of *Dnmt3l*, respectively, and those of three, three, and five mice for wild-type, heterozygous, and homozygous KO of *Pld6*, respectively. The mRNA libraries were produced using the NEBNext Ultra II Directional mRNA Library Prep Kit for Illumina (New England Biolabs). Sequences were obtained on a HiSeq2500 (Illumina) using a 50 bp single-end mode (Macrogen, Japan). The expression level of each mouse-specific retrotransposon subfamily was analyzed by mapping the reads onto the respective consensus sequences using hisat2 [17] as described previously [7].

### Whole-genome bisulfite sequencing and analysis (BS-seq)

Genomic DNA was prepared from wild-type and *Pld6* KO spermatogonia at P7 by phenol–chloroform extraction, fragmented by sonication on a Bioruptor (BMBio), and treated with bisulfite, as described previously [16]. The sequencing library was constructed using the ACCEL-NGS methyl-seq library kit (Swift Biosciences) and sequenced on a HiSeq Ten X (Illumina) using the 150 bp paired-end mode (Macrogen, Japan), yielding 120 and 200 million reads, which correspond to ∼7× and 11× coverage of the genome, respectively.

The obtained sequences were trimmed for adaptors and low-quality sequences using Trim_Galore! (https://www.bioinformatics.babraham.ac.uk/projects/trim_galore/) and the first 10 base pairs were removed. The trimmed reads were mapped onto the mouse genome (mm10) using Bismark [18]. The methylation calls at CpG sites were used to calculate the DNA methylation levels of individual retrotransposon loci using repeatmasker annotation downloaded from the UCSC table browser as well as L1 5′ regions identified in this study. Loci that had a total of ≥10 methylation calls were used for downstream analysis.

### Chromatin immunoprecipitation and sequencing and analysis (ChIP-seq)

Native chromatin was prepared as described previously [19] from spermatogonia at P7 (∼150 000 cells from three individuals for each genotype) and L/Z spermatocytes at P21 (∼200 000 cells from two individuals for each genotype). ChIP was performed against anti-H3K9me3 antibody (MABI0308, Monoclonal Research Institute) or anti-H3K4me3 antibody (07-473, Merck) for 2 h at 4°C using Dynabeads M280 sheep anti-mouse or rabbit IgG (ThermoFisher Scientific). Sequence libraries were prepared using the NEBNext Ultra DNA Library Prep Kit for Illumina (New England Biolab) and sequenced on a HiSeq Ten X (Illumina) using the 150 bp paired-end mode (Macrogen, Japan). The reads were trimmed using Trim_Galore!, and mapped onto the mouse genome (mm10) using hisat2. Uniquely mapped reads were used to calculate ChIP enrichment of individual retrotransposon loci.

### Identification and annotation of 5′ regions of genomic L1 copies

Using repeatmasker (www.repeatmasker.org) and a custom library containing only 5′ regions of mouse L1 subfamilies, L1 5′ regions were identified in the mm10 genome. The identified regions arranged consecutively were merged and re-annotated (Supplementary Table S2). They were intersected with the normal repeatmasker annotation table (downloaded from the UCSC table browser) using bedtools2 to confirm that they were partial L1 sequences.

## Results and discussion

### Some retrotransposons were up-regulated in *Pld6* and *Dnmt3l* KO spermatogonia

We performed mRNA-seq on wild-type, *Dnmt3l* KO, and *Pld6* KO mutant spermatogonia purified by FACS using an antibody against EpCAM on P7. Their overall gene expression profiles (Supplementary Fig. S2A) and marker gene expression (Supplementary Fig. S2B, C) were comparable with previously reported transcriptomes of spermatogonia. Although the EpCam-positive cells expressed *Kit*, a maker for differentiated spermatogonia, their transcriptomes showed the highest similarity to those of undifferentiated spermatogonia (OCT4-positive and ID4-high/low). There were up-regulated (186 and 59 in *Dnmt3l* and *Pld6 KO*, respectively; ≧4-fold, q < 0.05) and down-regulated (66 and 4 in *Dnmt3l* and *Pld6 KO*, respectively; ≧4-fold, q < 0.05) genes in the mutants, with no enrichment of Gene Ontology related to spermatogenesis (Supplementary Fig. S3). Moreover, the expression of spermatogonium-marker genes was largely unaffected (Supplementary Fig. S3E), indicating a comparable cell population between the wild-type and mutant samples.

In mice, L1 is a major LINE family, which is divided into subfamilies based on promoter sequences. In Repbase [20] and Dfam [21], their consensus sequences are divided into the 5′ region [containing a promoter and open reading frame 1 (ORF1)], ORF2, and the 3′ region. Mouse LTR elements include intracisternal A particles (IAPs), MMERVK10C, and MMERGLN, and the consensus sequences of the LTR region (containing a promoter) and internal region (encoding *gag, pol*, and *env* proteins) have been deposited separately. For example, the internal region of MMERVK10C is MMERVK10C-int, whereas its LTR region is RLTR10C. Here, we used these separated consensus sequences to calculate the subfamily-level expression. To this end, sequencing reads were mapped to the respective consensus sequences of mouse retrotransposons (see the Materials and methods).

**Figure 1.**
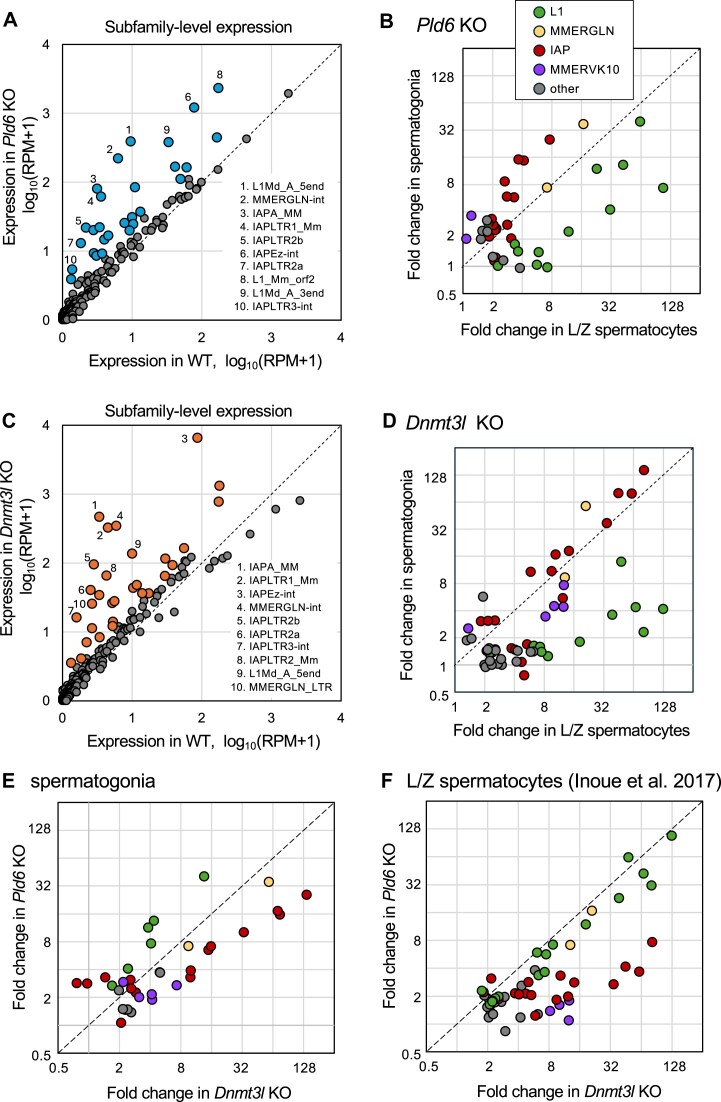
RNA expression of retrotransposons in the mutants. (**A**) Plot for retrotransposon expression in wild-type (WT) and *Pld6* KO spermatogonia at P7. The expression level is shown as reads per million mapped reads (RPM). Retrotransposons with > 2-fold up-regulation are highlighted in light blue. The top 10 up-regulated retrotransposons (RepBase repository) are indicated. (**B**) Fold changes in transcriptional increase in *Pld6* KO from the wild type are compared between spermatocytes at P21 and spermatogonia at P7. Fold changes were calculated as (RPM_mut_+ 1)/(RPM_wt_+ 1). Families of L1, MMERGLN, IAP, MMERVK10, and others are colored. Only retrotransposons with a > 2-fold increase by the mutation are shown. The data for spermatocytes were from Inoue *et al.* [7]. (**C**) Plot for retrotransposon expression in wild-type and *Dnmt3l* KO spermatogonia at P7. The expression level is shown as RPM. Retrotransposons with > 2-fold up-regulation are highlighted in orange. The top 10 up-regulated retrotransposons (RepBase repository) are indicated. (**D**) Fold changes in transcriptional increase in *Dnmt3l* KO from the wild type are compared between spermatocytes at P21 and spermatogonia at P7. The data for spermatocytes were from Inoue *et al.* [7]. (**E**) Fold changes in transcriptional increase in *Dnmt3l* KO and *Pld6* KO spermatogonia. (**F**) Fold changes in transcriptional increase in *Dnmt3l* KO and *Pld6* KO spermatocytes (data from [7]).

In the *Pld6* KO spermatogonia (Fig. 1A; Supplementary Table S1), L1Md_A_5end [the promoter, 5′ untranslated region (UTR), and ORF1 of L1Md_A] was up-regulated 40-fold. Other regions of L1, such as L1_Mm_orf2 and L1Md_A_3end, were also up-regulated 12-fold. L1Md_Gf_5end was up-regulated 7-fold. However, the degree of up-regulation of these L1 subfamilies was lower than that observed in *Pld6* KO spermatocytes (23- to 108-fold) (Fig. 1B). Evolutionarily young LTR retrotransposons, such as members of the IAP and MMERGLN families, were up-regulated up to 25- and 37-fold, respectively, in *Pld6* KO spermatogonia. It should be noted that in *Pld6* KO mutants, IAPEz-int and IAPLTR1_Mm (together constituting a full-length young IAP element) were up-regulated more in spermatogonia than in spermatocytes (Fig. 1B), suggesting that the piRNA system works more efficiently in spermatogonia than in spermatocytes. It is formally possible that transcription factors necessary for IAPEz expression are present at a lower level in spermatocytes than in spermatogonia. However, the similar levels of transcriptional activation in spermatogonia and spermatocytes in the *Dnmt3l* mutants (see below) argues against this. In contrast to IAPEz, MMERGLN expression was up-regulated to similar levels in *Pld6* KO spermatogonia and spermatocytes. This up-regulation may largely occur at the transcriptional level because MMERGLN elements exhibit loss of DNA methylation due to the *Pld6* KO mutation [7]. MMERVK10C and its LTR, LTR10C, were also up-regulated 2- to 3-fold by the *Pld6* KO mutation.

In *Dnmt3l* KO spermatogonia (Fig. 1C; Supplementary Table S1), IAP and MMERGLN were highly up-regulated (60- to 140-fold for IAPA_Mm, IAPEz-int, IAPLTR1_Mm, and MMERGLN-int). The degree of up-regulation of these LTR elements in *Dnmt3l* KO spermatogonia was similar to that observed in *Dnmt3l* KO spermatocytes (Fig. 1D) and higher than that in *Pld6* KO spermatogonia (Fig. 1E). These results suggest that in spermatogonia, the major mechanism for the regulation of LTR elements switches from the piRNA system to DNA methylation. Similarly, MMERVK10C was up-regulated 8-fold in *Dnmt3l* KO spermatogonia, which was higher than in *Pld6* KO spermatogonia. In contrast, L1 retrotransposons were only marginally up-regulated in *Dnmt3l* KO spermatogonia (4- to 14-fold for L1Md_A_5end, L1_Mm_orf2, L1Md_A_3end, and L1Md_Gf_5end). The degree of up-regulation was much lower than in *Dnmt3l* KO spermatocytes (39- to 127-fold) (Fig. 1D). Moreover, these L1s were less up-regulated in *Dnmt3l* KO spermatogonia than in *Pld6* KO spermatogonia (Fig. 1E), suggesting that the piRNA system effectively regulates the expression of L1 subfamilies in spermatogonia, even when the loss of DNA methylation at these L1s in *Pld6* KO spermatogonia [7] is taken into account.

Compared with the up-regulation in mutant spermatocytes (Fig. 1F), the fold increases in IAP and MMERVK10C expression in *Pld6* KO were higher in spermatogonia, whereas those in *Dnmt3l* KO were lower in spermatogonia. In both *Pld6* and *Dnmt3l* KO mice, the expression of L1 subfamilies increased from spermatogonia to spermatocytes. Therefore, the dependence of retrotransposon repression on DNA methylation became more evident as development progressed.

### Reduction of DNA methylation at individual retrotransposon copies

We previously showed the global and selective loss of DNA methylation in retrotransposon families in mutants [7]. However, the read depths were too shallow for the locus-level analyses. Here, we conducted whole-genome BS-seq of *Pld6* KO spermatogonia with more read numbers and longer read lengths than those in previous reports. High-coverage DNA methylation data for *Dnmt3l* KO spermatogonia were obtained from a previous study [22]. This allowed us to conduct the analysis at the locus level.

Promoter methylation is important for transcriptional regulation; therefore, the DNA methylation status of the L1 promoter regions should be analyzed separately from other parts of the L1 sequences. To this end, we identified L1 promoter regions that consisted of variable numbers of tandemly repeated monomers (Supplementary Table S2).

**Figure 2.**
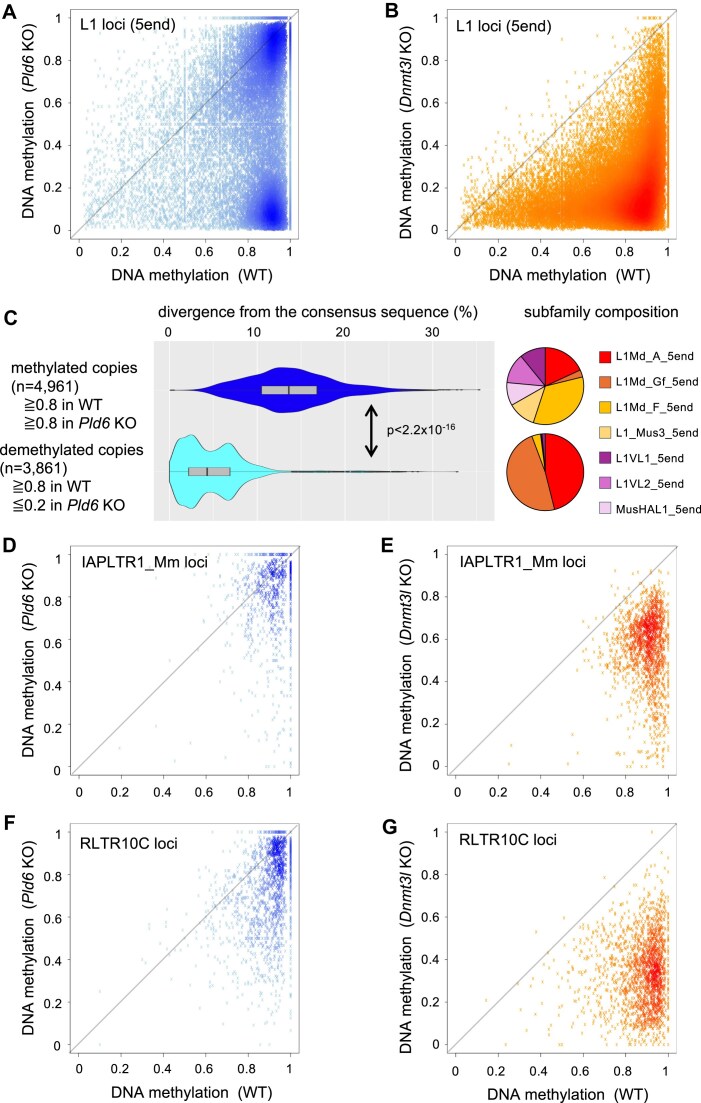
DNA methylation of retrotransposons in the mutants. (**A**) Plot showing DNA methylation levels at individual 5′ L1 sequences in wild-type (WT) and *Pld6* KO spermatogonia. (**B**) Plot showing DNA methylation levels at individual 5′ L1 sequences in wild-type and *Dnmt3l* KO spermatogonia. (**C**) Left: violin plots showing nucleotide divergence of *Pld6*-independent loci (blue) and *Pld6*-dependent loci (light blue). The *P*-value by U-test is shown. Right: pie charts showing the fraction of each subfamily included in *Pld6*-independent loci (top) and *Pld6*-dependent loci (bottom). (**D**) Plot showing DNA methylation levels at individual IAPLTR1_Mm sequences in wild-type and *Pld6* KO spermatogonia. (**E**) Plot showing DNA methylation levels at individual IAPLTR1_Mm sequences in wild-type and *Dnmt3l* KO spermatogonia. (**F**) Plot showing DNA methylation levels at individual RLTR10C sequences in wild-type and *Pld6* KO spermatogonia. (**G**) Plot showing DNA methylation levels at individual RLTR10C sequences in wild-type and *Dnmt3l* KO spermatogonia.

Locus-level analysis of L1 promoter methylation revealed that, whereas methylation of most L1 copies was reduced in the *Dnmt3l* KO mutants, there were two major populations in the *Pld6* KO mutants (Fig. 2A, B): (i) highly methylated in both the wild type and *Pld6* KO mutants, and (ii) highly methylated in the wild type but hypomethylated in the *Pld6* KO mutants. The hypomethylated copies (*Pld6*-dependent copies) were mostly evolutionarily younger L1Md_A and L1Md_Gf subfamilies that showed low sequence divergence from their respective consensus sequences, whereas older copies were hypermethylated (Fig. 2C, *P*< 2.2 × 10^−16^ by U-test). Even among the L1Md_A and L1Md_Gf subfamilies, older copies were hypermethylated (Supplementary Fig. S4). It was recently reported that SPOCD1 and SPIN1 mark the promoter regions of young L1 subfamilies before PIWIL4 mediates piRNA-directed DNA methylation. The *Spocd1* mutation results in hypomethylation of young L1 subfamilies, but not of old subfamilies [23] similar to the *Pld6* KO mutation. These results suggest that the SPOCD1–SPIN1–piRNA system efficiently targets young L1 promoters for DNA methylation. In contrast, *Pld6* KO mutation did not severely decrease DNA methylation in copies of IAPLTR1_Mm (constituting the promoter of IAPEz-int) or RLTR10C (constituting the promoter of MMERVK10C-int) (Fig. 2D, F). We noted that, although few, some IAPLTR1 and RLTR10C copies were demethylated, suggesting locus-specific, piRNA-directed *de novo* DNA methylation of these retrotransposon copies, as observed previously [14, 24, 25]. In the *Dnmt3l* KO methylome, IAPLTR1_Mm was mildly demethylated compared with the wild-type methylomes, although some loci were severely demethylated (Fig. 2E). In contrast, RLTR10C was severely demethylated by the *Dnmt3l* KO mutation in general (Fig. 2G).

**Figure 3.**
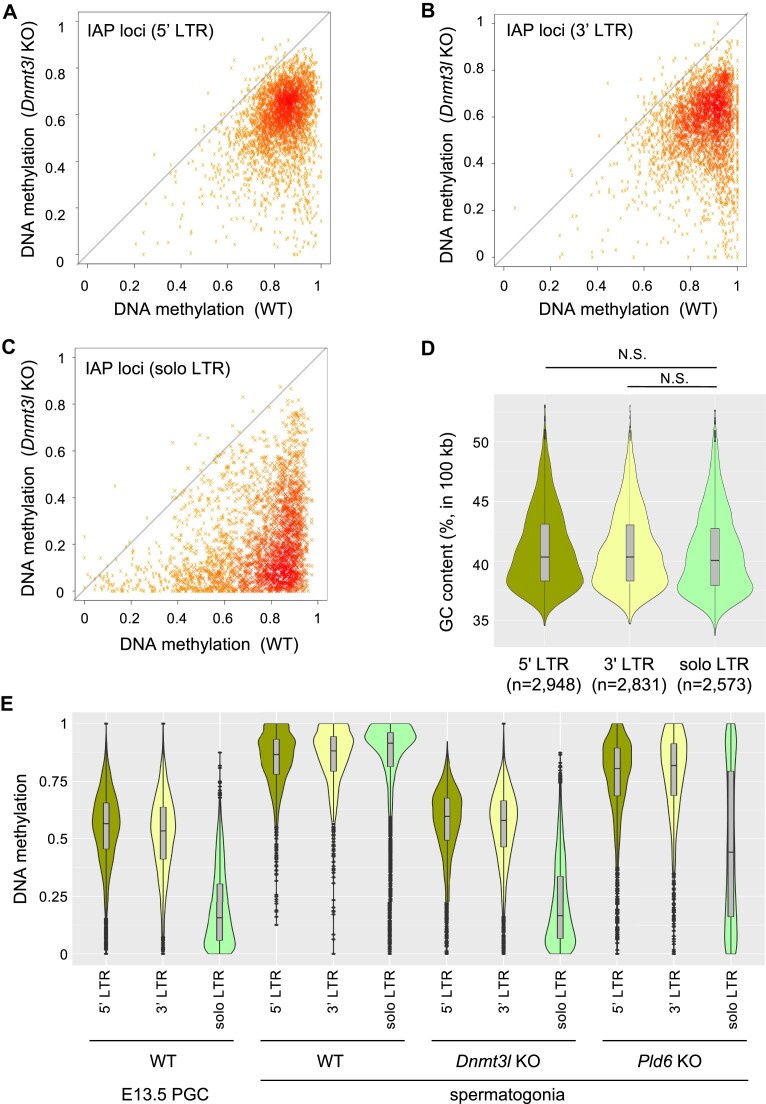
DNA methylation of IAP LTR sequences. (**A**–**C**) Plot showing DNA methylation levels at individual IAP LTR sequences in wild-type (WT) and *Dnmt3l* KO spermatogonia for the 5′ LTR (A), 3′ LTR (B), and solo LTR (C). (**D**) Violin plot showing genomic GC content in the neighboring regions of IAP LTR loci (50 kb upstream and 50 kb downstream). N.S., not significant by U-tests. (**E**) Violin plots showing DNA methylation levels of 5′, 3′, and solo LTRs of IAP in PGCs and spermatogonia.

To elucidate the locus-dependent methylation loss in IAP LTRs, the loci were categorized into three groups, 5′ LTR, 3′ LTR, and solo LTR, by referring to our previous report [26]. This revealed that 5′ and 3′ LTRs were highly methylated in both wild-type and *Dnmt3l* KO spermatogonia (Fig. 3A, B), whereas solo LTRs were severely demethylated by the *Dnmt3l* KO mutation (Fig. 3C). The *Dnmt3l* dependency of DNA methylation was dictated by the local GC content in the genome [7]; therefore, we checked the GC contents of neighboring regions (50 kb upstream and downstream) and found no significant difference between full-length IAP-associated LTRs and solo LTRs (Fig. 3D). Since IAPs are known to be resistant to genome-wide DNA demethylation in PGCs [27, 28], we analyzed their DNA methylation status in E13.5 wild-type PGCs using published whole-genome BS-seq data [29]. The 5′ and 3′ LTR copies were highly methylated in PGCs, while solo LTRs were hypomethylated (Fig. 3E). In *Dnmt3l* KO spermatogonia, DNA methylation levels were almost the same as those in PGCs (Fig. 3E), suggesting little or no *de novo* methylation at the IAP loci. Therefore, variation in demethylation levels in PGCs explains the variable levels of DNA methylation in *Dnmt3l* KO spermatogonia. The internal IAP sequence (IAPEZ-int) may confer resistance to genome-wide demethylation in PGCs. Similar to human PGCs [30], it is conceivable that Krüppel-associated box zinc finger proteins (KRAB-ZFPs) bind to the internal IAP sequence and protect both the internal and LTR sequences from demethylation. KRAB-ZFPs are known to recruit SETDB1 [31], which leads to H3K9me3 deposition at the sites of their binding [9, 10], and in turn maintains DNA methylation, given that UHRF1, an essential cofactor for DNMT1 [32], binds to H3K9me3-marked regions [33].

### Loss of DNA methylation decreased H3K9me3 at evolutionarily young L1 subfamilies

**Figure 4.**
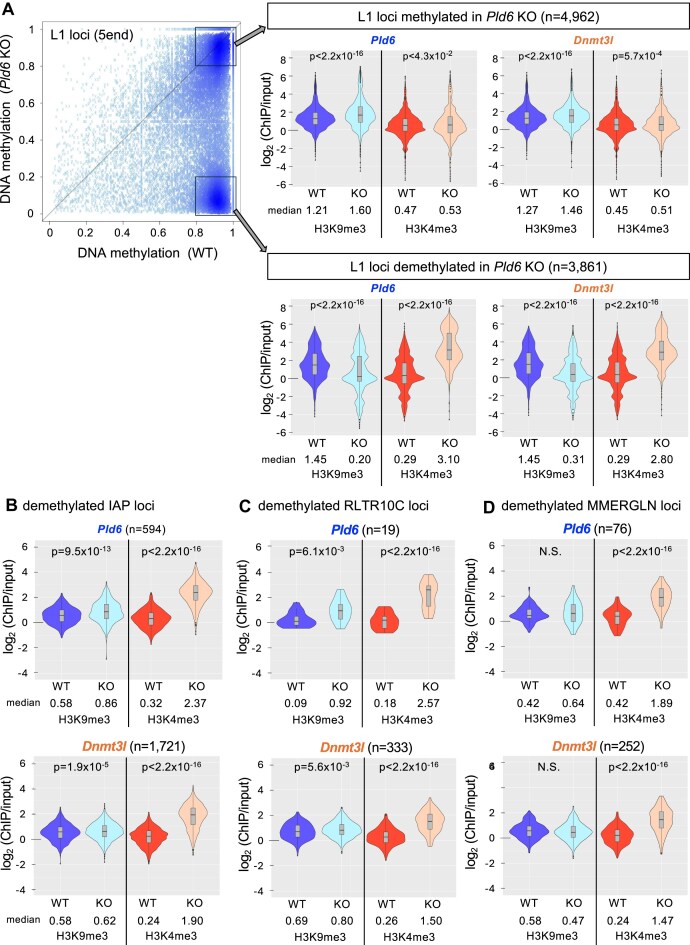
H3K9me3 and H3K4me3 at retrotransposon loci in the mutant spermatogonia. (**A**) Violin plots showing log_2_ ChIP enrichment of H3K9me3 and H3K4me3 at *Pld6*-independent loci (top) and *Pld6*-dependent loci (bottom) in *Pld6* and *Dnmt3l* KO spermatogonia. (**B**) Violin plots showing ChIP enrichment for H3K9me3 and H3K4me3 at IAP LTR loci demethylated in *Pld6* KO (top) and *Dnmt3l* KO spermatogonia (bottom). (**C**) Violin plots showing ChIP enrichment for H3K9me3 and H3K4me3 at the demethylated RLTR10C loci in *Pld6 KO* (top) and *Dnmt3l* KO spermatogonia (bottom). (**D**) Violin plots showing ChIP enrichment for H3K9me3 and H3K4me3 at the demethylated MMERGLN loci in *Pld6 KO* (top) and *Dnmt3l* KO spermatogonia (bottom). For all comparisons between the wild type (WT) and KO, *P*-values were calculated by U-tests (note: the lowest *P*-value is 2.2 × 10^−16^ when calculated by R).

**Figure 5.**
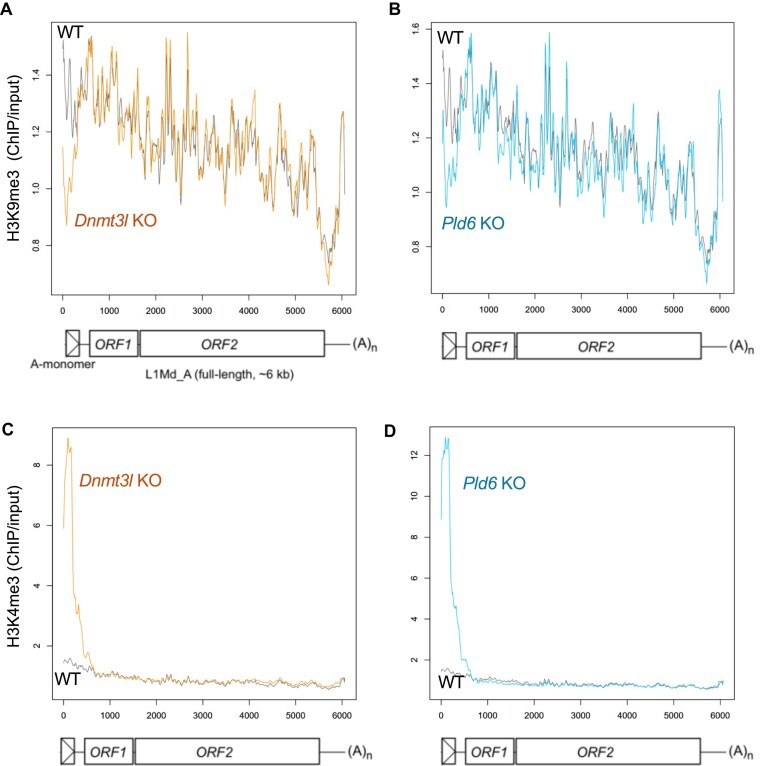
H3K9me3 and H3K4me3 levels in the L1 body (subfamily-level analysis). ChIP-seq reads were mapped onto the full-length consensus sequence of the L1Md_A family (with a single copy of A-monomer). Fold enrichments of ChIPseq reads relative to input reads (in 50 bp bins) were plotted along the L1 sequence for H3K9me3 in *Dnmt3l* KO (**A**), H3K9me3 in *Pld6* KO (**B**), H3K4me3 in *Dnmt3l* KO (**C**), and H3K4me3 in *Pld6* KO (**D**). Gray lines, wild type; orange lines, *Dnmt3l* KO; light blue lines, *Pld6* KO.

**Figure 6.**
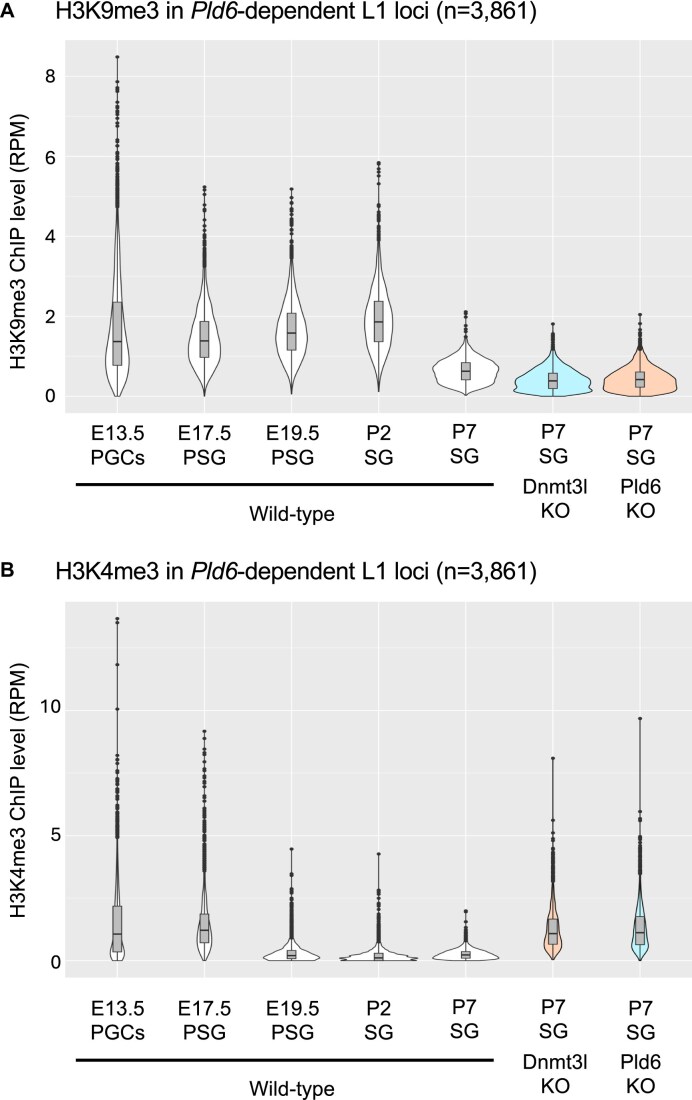
Histone methylation at *Pld6*-dependent L1 copies during development. (**A**) Dynamics of the H3K9me3 level at *Pld6*-dependent L1 copies. The ChIP-seq data for E13.5 to P2 were obtained from the Gene Expression Omnibus (GEO; GSE121118). The P7 data were generated in this study. The H3K9me3 level was kept high from E13.6 to P2. Note that different antibodies were used in the two experiments (E13.5-to-P2 versus P7), thus direct comparison is not applicable. PGCs, primordial germ cells; PSG, prospermatogonia; SG, spermatogonia. (**B**) Dynamics of the H3K4me3 level. The ChIP-seq data for E13.5 to P2 were obtained from the GEO (GSE121118). The P7 data were generated in this study. The H3K4me3 level was high at E13.5, then gradually declined as the cells developed.

H3K9me3 has been reported to be involved in retrotransposon regulation in PGCs and spermatocytes [9, 10]. Moreover, the failure in the piRNA pathway in prospermatogonia leads to a loss of H3K9me3 in the L1 promoter [5]. To examine whether H3K9me3 was compromised in the two mutants, ChIP-seq of H3K9me3 was performed using wild-type and mutant spermatogonia (Fig. 4; Supplementary Table S1). Analysis of the L1 sequences revealed that H3K9me3 was decreased in both mutants in the L1 promoter region but not in its body (Fig. 5A, B). As shown above, DNA methylation at L1 promoters is differentially affected depending on evolutionary age. Therefore, we separately analyzed the two L1 populations, *Pld6*-dependent and -independent loci for DNA methylation (Fig. 4A). In the wild type, both types of L1 loci were enriched with H3K9me3 at similar levels. *Pld6*-independent loci (DNA methylated in *Pld6* KO) did not show significant changes in H3K9me3, whereas *Pld6*-dependent loci showed a large decrease (>2-fold) in *Pld6* KO spermatogonia. These results are consistent with the idea that piRNAs guide H3K9me3 in young L1 promoters [5]. However, it is unclear whether this is a direct effect because piRNAs also direct DNA methylation. To address this, we analyzed H3K9me3 in *Dnmt3l* mutants in which *de novo* DNA methylation, but not the piRNA system, was disrupted. A marked reduction in H3K9me3 was observed at *Pld6*-dependent loci (Fig. 4A). As young L1s were marked with H3K9me3 at the PGC and later stages [9, 34] (Fig. 6A), it is likely that the H3K9me3 decrease was due to its loss rather than a failure in initial deposition. Collectively, these results suggest that DNA methylation is important for the maintenance of H3K9me3 in young L1 promoters during male germ cell development.

In contrast, the LTRs of IAP (Fig. 4B), MMERVK10C (Fig. 4C), and MMERGLN (Fig. 4D), which showed decreased DNA methylation in *Pld6* or *Dnmt3l* KO spermatogonia did not show a decrease in H3K9me3. A possibility is that DNA methylation-insensitive KRAB-ZFPs bind to these retrotransposon loci and maintain the H3K9me3 levels. Collectively, the *de novo* DNA methylation-dependent maintenance of H3K9me3 is not a universal feature of young retrotransposons, but is rather particularly important for young L1 elements.

Regarding H3K9me3 at L1 promoters, it is conceivable that a methylation-dependent, sequence-specific DNA-binding protein(s) binds to L1 promoters and recruits H3K9 methyltransferases. For example, ZFP57, one of the KRAB-ZFPs, binds specifically to methylated DNA and serves as a scaffold for the repressive complex. However, ZFP57 does not bind to L1 sequences. Moreover, the removal of ZFP57 protein or genome-wide DNA methylation in mouse embryonic stem cells (ESCs) does not affect the H3K9me3 level at LTR elements including ZFP57-bound elements [35, 36]. To gain insights, we analyzed published ChIP-seq data for 65 KRAB-ZFPs in mouse ESCs [37], which revealed that four of them, Gm114412, Gm13152, Gm14419, and Zfp961, bind strongly or marginally to the 5′ region of *Pld6*-dependent loci, whereas their binding was very weak to *Pld6-*independent loci (Supplementary Fig. S5A). However, the H3K9me3 levels in the L1 loci were not reduced in the absence of DNA methylation [36] (Supplementary Fig. S5B), suggesting DNA methylation-independent maintenance of H3K9me3 in ESCs. It would be interesting to identify a protein that transfers the information of DNA methylation to the histone modification states at young L1 loci in germ cells.

**Figure 7.**
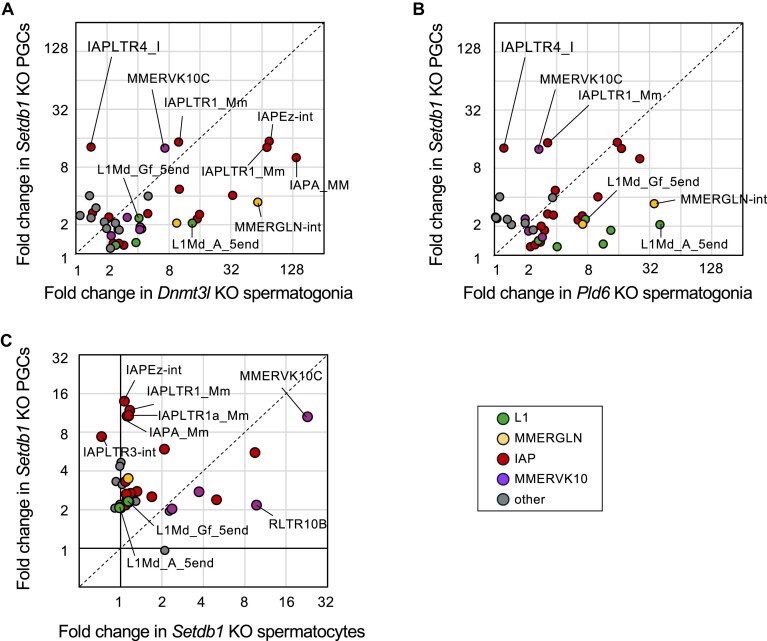
Comparison of effects of *Setdb1, Dnmt3l*, and *Pld6* KO mutations (subfamily-level analysis). (**A**) Plot of fold changes in retrotransposon expression in *Dnmt3l* KO and *Setdb1* KO mutants. (**B**) Plot of fold changes in retrotransposon expression in *Pld6* KO and *Setdb1* KO mutants. (**C**) Plot of fold changes in retrotransposon expression in *Setdb1* KO spermatocytes and PGCs. Retrotransposons are colored by their families. mRNA-seq data were obtained from the GEO (GSE60377 for *Setdb1* KO PGCs and GSE107671for *Setdb1* KO spermatocytes).

Regarding the importance of DNA methylation versus H3K9me3, we compared the published results of *Setdb1* KO PGCs [9] and spermatocytes [10] with the results in this study (Fig. 7). In most cases, including IAPEz-int and L1Md_A_5end, higher up-regulation was seen in *Dnmt3l* KO spermatogonia than in*Setdb1* KO PGCs (Fig. 7A). When *Pld6* KO spermatogonia were compared with *Setdb1* KO PGCs (Fig. 7B), the retrotransposons showing loss of DNA methylation (L1Md_A_5end, L1Md_Gf_5end, and MMERGLN) were more up-regulated in *Pld6* KO spermatogonia, while IAPLTR1_Mm and MMERVK10C were more up-regulated in *Setdb1* KO PGCs. Comparison of PGCs and spermatocytes of *Setdb1* KO mutants revealed that transcriptional up-regulation of many retrotransposons was higher in PGCs than in spermatocytes (Fig. 7C), suggesting that the relative importance of H3K9me3 in retrotransposon repression is decreased in later developmental stages. These results indicate that during male germ cell development, DNA methylation plays a more important role than H3K9me3 in silencing of specific retrotransposons, including young L1 families.

### Loss of DNA methylation increased H3K4me3 at retrotransposons

Next, we performed ChIP-seq for active H3K4me3 marks in wild-type and mutant spermatogonia (Fig. 4; Supplementary Table S1). Analysis of the L1 sequences revealed that H3K4me3 increased in both mutants in the promoter region (Fig. 5C, D). At *Pld6-*independent L1 loci, H3K4me3 was unchanged in *Pld6* KO mutants (Fig. 4A). The level of H3K4me3 was unchanged even in the *Dnmt3l* mutants, where DNA methylation was lost at these loci. At the *Pld6-*dependent L1 loci, H3K4me3 was increased by ∼10-fold in both mutants (*P*< 2.2 × 10^−16^ by U-tests), which is consistent with previous ChIP-qPCR results in the *Dnmt3l* mutants [22]. The H3K4me3 levels at these loci were high at E13.5, then became very low at E19.5 [34] (Fig. 6B), suggesting that DNA methylation is important for maintaining the low level of H3K4me3 at these loci in spermatogonia. Alternatively, the simultaneous occurrence of the H3K4me3 loss and the DNA methylation gain in these loci in prospermatogonia suggests that DNA methylation may direct H3K4 demethylation. In the latter case, the persistent H3K4me3 in these loci could result in a failure in H3K9me3 deposition, given that H3K4me3 inhibits the activity of H3K9 methyltransferases including SETDB1 [38].

At LTR retrotransposons, loci with decreased DNA methylation showed an increase in H3K4me3 in both mutants by ∼4-fold (Fig. 4B, C, and D for IAP, MMERVK10C, and MMERGLN, respectively), which is in stark contrast to the slight changes in H3K9me3. Again, it is suggested that DNA methylation of retrotransposon promoters counteracts H3K4me3. The increase in H3K4me3 in the mutants was probably related to the transcriptional up-regulation of these retrotransposons.

### The level of H3K9me3 in retrotransposons decreased in spermatocytes

**Figure 8.**
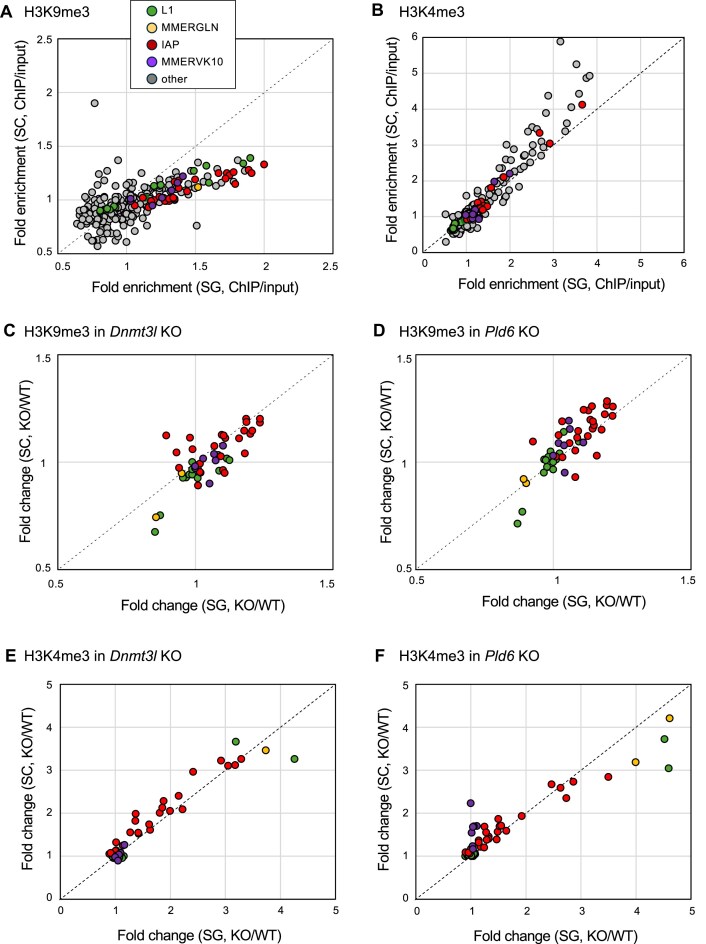
H3K9me3 and H3K4me3 levels in P7 spermatogonia and P21 spermatocytes (subfamily-level analysis). (**A**) Fold enrichments (ChIP/input) of H3K9me3 at retrotransposon families in spermatogonia (*x*-axis) and spermatocytes (*y*-axis). The dashed line indicates the *y* = *x* slope. Colors indicate retrotransposon families shown in the insert (SG, spermatogonia; SC, spermatocytes). (**B**) Fold enrichments (ChIP/input) of H3K4me3 at retrotransposon families in spermatogonia (*x*-axis) and spermatocytes (*y*-axis). The dashed line indicates the *y* = *x* slope. (**C**) Fold changes in H3K9me3 at retrotransposons (excluding “others”) in spermatogonia (*x*-axis) and spermatocytes (*y*-axis) in *Dnmt3l* KO. (**D**) Fold changes in H3K9me3 at retrotransposons (excluding “others”) in spermatogonia (*x*-axis) and spermatocytes (*y*-axis) in *Pld6* KO. (**E**) Fold changes in H3K4me3 at retrotransposons (excluding “others”) in spermatogonia (*x*-axis) and spermatocytes (*y*-axis) in *Dnmt3l* KO. (**F**) Fold changes in H3K4me3 at retrotransposons (excluding “others”) in spermatogonia (*x*-axis) and spermatocytes (*y*-axis) in *Pld6* KO.

To compare histone modifications between spermatogonia and spermatocytes, we performed ChIP-seq for H3K4me3 and H3K9me3 in the L/Z stages of spermatocytes purified by FACS. In the wild type, retrotransposons enriched with H3K9me3 in spermatogonia, such as L1Md_A_5end and IAPLTR1_Mm, showed reduced H3K9me3 levels in spermatocytes (Fig. 8A), and retrotransposons enriched with H3K4me3 in spermatogonia showed slightly increased H3K4me3 levels in spermatocytes (Fig. 8B). The reduced H3K9me3 levels would account for the increased retrotransposon expression in *Dnmt3l* KO and *Pld6* KO spermatocytes despite the fact that fold changes of H3K9me3 and H3K4me3 in the mutants were comparable between spermatogonia and spermatocytes (Fig. 8C–F). The fact that H3K9me3 is reduced from spermatogonia to spermatocytes is consistent with the idea that DNA methylation is a pivotal epigenetic modification for retrotransposon regulation in spermatocytes.

## Conclusion

In this study, we investigated how piRNA deficiency and failure of *de novo* DNA methylation in mouse male germ cells affect RNA expression, DNA methylation, H3K9me3, and H3K4me3 at retrotransposon loci. These results reveal that DNA methylation is important for maintaining high H3K9me3 and low H3K4me3 levels in young L1 promoters to inhibit active L1 transcription. piRNAs induce *de novo* DNA methylation at young L1 loci, which in turn maintains the H3K9me3 level. Among LTR elements, H3K4me3 and its associated transcription increased when DNA methylation decreased, whereas H3K9me3 was not affected. Thus, it should also be noted that the H3K9me3 landscape in spermatogonia is largely determined by other factor(s), while H3K9me3 at young L1 loci is maintained in a DNA methylation-dependent manner. DNA methylation is also important for suppressing H3K4me3 in many retrotransposons in male germ cells.

An increasing number of studies have reported regulatory interactions between histone modifications and DNA methylation in mammalian systems. For example, in mouse prospermatogonia, methylation at H3K36 and demethylation at H3K4 direct *de novo* DNA methylation [11, 12, 39]. In mouse ESCs, H3K9me2/3 and H4K20me3 maintain DNA hypermethylation in retrotransposons [40–42]. However, loss of DNA methylation does not reduce H3K9me3 at retrotransposons [36, 42], although a limited number of unique regions including imprinting control regions show DNA methylation-dependent H3K9me3 through binding by ZFP57 [35]. Taken together, DNA methylation appears to act downstream of the regulatory pathways of epigenetic modifications at retrotransposons. However, the results of this study highlight that, in spermatogenic cells, DNA methylation is important for the regulation of histone modifications in a variety of retrotransposons, implying that DNA methylation is pivotal for retrotransposon silencing.

Histones H3.1, H3.2, and H3.3 are present in spermatogonia, whereas in spermatocytes, H3.1 and H3.2 are replaced by H3t, which reduces nucleosome stability [43, 44]. This histone variant exchange, specifically occurring at the spermatogonium–spermatocyte transition, may explain why DNA methylation is more important for the transcriptional silencing of retrotransposons in spermatocytes. In addition, the reduction of H3K9me3 in many retrotransposons in spermatocytes also seems linked to the change to DNA methylation-centered regulation. Moreover, the vast majority of histone nucleosomes are removed at the spermatid stage and eventually exchanged with protamine. Cells must repress retrotransposons during histone–protamine exchange, where DNA is the most reliable source of epigenetic modifications. The regulatory switch toward the DNA methylation-centered system may gradually occur in male germ cells in preparation for this future situation.

## Supplementary Material

gkaf1240_Supplemental_Files

## Data Availability

The BS-seq, mRNA-seq, and ChIP-seq data were deposited in the Gene Expression Omnibus under the accession numbers GSE296267, GSE296268, and GSE296269, respectively.

## Notes

Present address: Cellular Memory Laboratory, RIKEN Pioneering Research Institute, Wako, 351-0198, Japan

## References

[1] Kawase M, Ichiyanagi K. Mouse retrotransposons: sequence structure, evolutionary age, genomic distribution and function. Genes Genet Syst. 2024;98:337–51. 10.1266/ggs.23-00221. 37989301

[2] Sasaki H, Matsui Y. Epigenetic events in mammalian germ-cell development: reprogramming and beyond. Nat Rev Genet. 2008;9:129–40. 10.1038/nrg2295. 18197165

[3] Kawase M, Ichiyanagi K. The expression dynamics of piRNAs derived from male germline piRNA clusters and retrotransposons. Front Cell Dev Biol. 2022;10:868746. 10.3389/fcell.2022.868746. 35646920 PMC9130748

[4] Chuma S, Nakano T. piRNA and spermatogenesis in mice. Philos Trans R Soc B: Biol Sci. 2013;368:20110338. 10.1098/rstb.2011.0338. PMC3539364 23166399

[5] Pezic D, Manakov SA, Sachidanandam R et al. piRNA pathway targets active LINE1 elements to establish the repressive H3K9me3 mark in germ cells. Genes Dev. 2014;28:1410–28. 10.1101/gad.240895.114. 24939875 PMC4083086

[6] Reuter M, Berninger P, Chuma S et al. Miwi catalysis is required for piRNA amplification-independent LINE1 transposon silencing. Nature. 2011;480:264–7. 10.1038/nature10672. 22121019

[7] Inoue K, Ichiyanagi K, Fukuda K. et al. Switching of dominant retrotransposon silencing strategies from posttranscriptional to transcriptional mechanisms during male germ-cell development in mice. PLoS Genet. 2017;13:e1006926. 10.1371/journal.pgen.1006926. 28749988 PMC5549759

[8] Bourc’his D, Bestor TH. Meiotic catastrophe and retrotransposon reactivation in male germ cells lacking Dnmt3L. Nature. 2004;431:96–9. 10.1038/nature02886. 15318244

[9] Liu S, Brind’Amour J, Karimi MM et al. Setdb1 is required for germline development and silencing of H3K9me3-marked endogenous retroviruses in primordial germ cells. Genes Dev. 2014;28:2041–55. 10.1101/gad.244848.114. 25228647 PMC4173156

[10] Hirota T, Blakeley P, Sangrithi MN et al. SETDB1 links the meiotic DNA damage response to sex chromosome silencing in mice. Dev Cell. 2018;47:645–59. 10.1016/j.devcel.2018.10.004. 30393076 PMC6286383

[11] Singh P, Li AX, Tran DA et al. De novo DNA methylation in the male germ line occurs by default but is excluded at sites of H3K4 methylation. Cell Rep. 2013;4:205–19. 10.1016/j.celrep.2013.06.004. 23810559 PMC3740941

[12] Nagamori I, Kobayashi H, Nishimura T et al. Relationship between PIWIL4-mediated H3K4me2 demethylation and piRNA-dependent DNA methylation. Cell Rep. 2018;25:350–6. 10.1016/j.celrep.2018.09.017. 30304676

[13] Hata K, Okano M, Lei H et al. Dnmt3L cooperates with the Dnmt3 family of de novo DNA methyltransferases to establish maternal imprints in mice. Development. 2002;129:1983–93. 10.1242/dev.129.8.1983. 11934864

[14] Watanabe T, Chuma S, Yamamoto Y et al. MITOPLD is a mitochondrial protein essential for nuage formation and piRNA biogenesis in the mouse germline. Dev Cell. 2011;20:364–75. 10.1016/j.devcel.2011.01.005. 21397847 PMC3062204

[15] Anderson R Schaible K, Heasman J et al. Expression of the homophilic adhesion molecule, Ep-CAM, in the mammalian germ line. J Reprod Fertil. 1999;116:379–84. 10615264 10.1530/jrf.0.1160379

[16] Ichiyanagi K, Li Y, Watanabe T et al. Locus- and domain-dependent control of DNA methylation at mouse B1 retrotransposons during male germ cell development. Genome Res. 2011;21:2058–66. 10.1101/gr.123679.111. 22042642 PMC3227096

[17] Kim D, Paggi JM, Park C et al. Graph-based genome alignment and genotyping with HISAT2 and HISAT-genotype. Nat Biotechnol. 2019;37:907–15. 10.1038/s41587-019-0201-4. 31375807 PMC7605509

[18] Krueger F, Andrews SR. Bismark: a flexible aligner and methylation caller for Bisulfite-Seq applications. Bioinformatics. 2011;27:1571–2. 10.1093/bioinformatics/btr167. 21493656 PMC3102221

[19] Hirata M, Ichiyanagi T, Katoh H et al. Sequence divergence and retrotransposon insertion underlie interspecific epigenetic differences in primates. Mol Biol Evol. 2022;39::msac208 10.1093/molbev/msac208. 36219870 PMC9577543

[20] Bao W, Kojima KK, Kohany O. Repbase Update, a database of repetitive elements in eukaryotic genomes. Mobile DNA. 2015;6:11. 10.1186/s13100-015-0041-9. 26045719 PMC4455052

[21] Storer J, Hubley R, Rosen J et al. The Dfam community resource of transposable element families, sequence models, and genome annotations. Mobile DNA. 2021;12:2. 10.1186/s13100-020-00230-y. 33436076 PMC7805219

[22] Zamudio N, Barau J, Teissandier A et al. DNA methylation restrains transposons from adopting a chromatin signature permissive for meiotic recombination. Genes Dev. 2015;29:1256–70. 10.1101/gad.257840.114. 26109049 PMC4495397

[23] Dias Mirandela M, Zoch A, Leismann J et al. Two-factor authentication underpins the precision of the piRNA pathway. Nature. 2024;634:979–85. 10.1038/s41586-024-07963-3. 39294378 PMC11499256

[24] Kuramochi-Miyagawa S, Watanabe T, Gotoh K et al. DNA methylation of retrotransposon genes is regulated by Piwi family members MILI and MIWI2 in murine fetal testes. Genes Dev. 2008;22:908–17. 10.1101/gad.1640708. 18381894 PMC2279202

[25] Molaro A, Falciatori I, Hodges E et al. Two waves of de novo methylation during mouse germ cell development. Genes Dev. 2014;28:1544–9. 10.1101/gad.244350.114. 25030694 PMC4102761

[26] Shimosuga KI, Fukuda K, Sasaki H et al. Locus-specific hypomethylation of the mouse IAP retrotransposon is associated with transcription factor-binding sites. Mobile DNA. 2017;8:20. 10.1186/s13100-017-0105-0. 29255492 PMC5729234

[27] Hajkova P, Erhardt S, Lane N et al. Epigenetic reprogramming in mouse primordial germ cells. Mech Dev. 2002;117:15–23. 10.1016/S0925-4773(02)00181-8. 12204247

[28] Lane N, Dean W, Erhardt S et al. Resistance of IAPs to methylation reprogramming may provide a mechanism for epigenetic inheritance in the mouse. Genesis. 2003;35:88–93. 10.1002/gene.10168. 12533790

[29] Kobayashi H, Sakurai T, Miura F et al. High-resolution DNA methylome analysis of primordial germ cells identifies gender-specific reprogramming in mice. Genome Res. 2013;23:616–27. 10.1101/gr.148023.112. 23410886 PMC3613579

[30] Fukuda K, Makino Y, Kaneko S et al. Potential role of KRAB-ZFP binding and transcriptional states on DNA methylation of retroelements in human male germ cells. eLife. 2022;11:e76822. 10.7554/eLife.76822. 35315771 PMC8967385

[31] Bruno M, Mahgoub M, Macfarlan TS. The arms race between KRAB-zinc finger proteins and endogenous retroelements and its impact on mammals. Annu Rev Genet. 2019;53:393–416. 10.1146/annurev-genet-112618-043717. 31518518

[32] Sharif J, Muto M, Takebayashi S et al. The SRA protein Np95 mediates epigenetic inheritance by recruiting Dnmt1 to methylated DNA. Nature. 2007;450:908–12. 10.1038/nature06397. 17994007

[33] Rottach A, Frauer C, Pichler G et al. The multi-domain protein Np95 connects DNA methylation and histone modification. Nucleic Acids Res. 2010;38:1796–804. 10.1093/nar/gkp1152. 20026581 PMC2847221

[34] Yamanaka S, Nishihara H, Toh H et al. Broad heterochromatic domains open in gonocyte development prior to de novo DNA methylation. Dev Cell. 2019;51:21–34.e5. 10.1016/j.devcel.2019.07.023e25. 31474564

[35] Shi H, Strogantsev R, Takahashi N et al. ZFP57 regulation of transposable elements and gene expression within and beyond imprinted domains. Epigenetics Chromatin. 2019;12:49. 10.1186/s13072-019-0295-4. 31399135 PMC6688207

[36] Karimi MM, Goyal P, Maksakova IA et al. DNA methylation and SETDB1/H3K9me3 regulate predominantly distinct sets of genes, retroelements, and chimeric transcripts in mESCs. Cell Stem Cell. 2011;8:676–87. 10.1016/j.stem.2011.04.004. 21624812 PMC3857791

[37] Wolf G, de Iaco A, Sun MA et al. KRAB-zinc finger protein gene expansion in response to active retrotransposons in the murine lineage. eLife. 2020;9::e56337 10.7554/eLife.56337. 32479262 PMC7289599

[38] Binda O, LeRoy G, Bua DJ et al. Trimethylation of histone H3 lysine 4 impairs methylation of histone H3 lysine 9: regulation of lysine methyltransferases by physical interaction with their substrates. Epigenetics. 2010;5:767–75. 10.4161/epi.5.8.13278. 21124070 PMC3052887

[39] Shirane K, Miura F, Ito T et al. NSD1-deposited H3K36me2 directs de novo methylation in the mouse male germline and counteracts Polycomb-associated silencing. Nat Genet. 2020;52:1088–98. 10.1038/s41588-020-0689-z. 32929285

[40] Ren W, Fan H, Grimm SA et al. DNMT1 reads heterochromatic H4K20me3 to reinforce LINE-1 DNA methylation. Nat Commun. 2021;12:2490. 10.1038/s41467-021-22665-4. 33941775 PMC8093215

[41] Wang Q, Yu G, Ming X et al. Imprecise DNMT1 activity coupled with neighbor-guided correction enables robust yet flexible epigenetic inheritance. Nat Genet. 2020;52:828–39. 10.1038/s41588-020-0661-y. 32690947

[42] Lehnertz B, Ueda Y, Derijck AA et al. Suv39h-mediated histone H3 lysine 9 methylation directs DNA methylation to major satellite repeats at pericentric heterochromatin. Curr Biol. 2003;13:1192–200. 10.1016/S0960-9822(03)00432-9. 12867029

[43] Ueda J, Harada A, Urahama T et al. Testis-specific histone variant H3t gene is essential for entry into spermatogenesis. Cell Rep. 2017;18:593–600. 10.1016/j.celrep.2016.12.065. 28099840

[44] Tachiwana H, Kagawa W, Osakabe A et al. Structural basis of instability of the nucleosome containing a testis-specific histone variant, human H3T. Proc Natl Acad Sci USA. 2010;107:10454–9. 10.1073/pnas.1003064107. 20498094 PMC2890842

