# Supplementary material for: DNA methylation dictates histone modifications in developing male germ cells in the mouse
Source: Nucleic Acids Res. 2025 Nov 20;53(21):gkaf1240. doi: 10.1093/nar/gkaf1240 (PMC12631079; doi:10.1093/nar/gkaf1240)
Supplement: gkaf1240_Supplemental_Files [file gkaf1240_supplemental_files.zip › Sugimoto_Supplementary_Figures_revised.pdf]

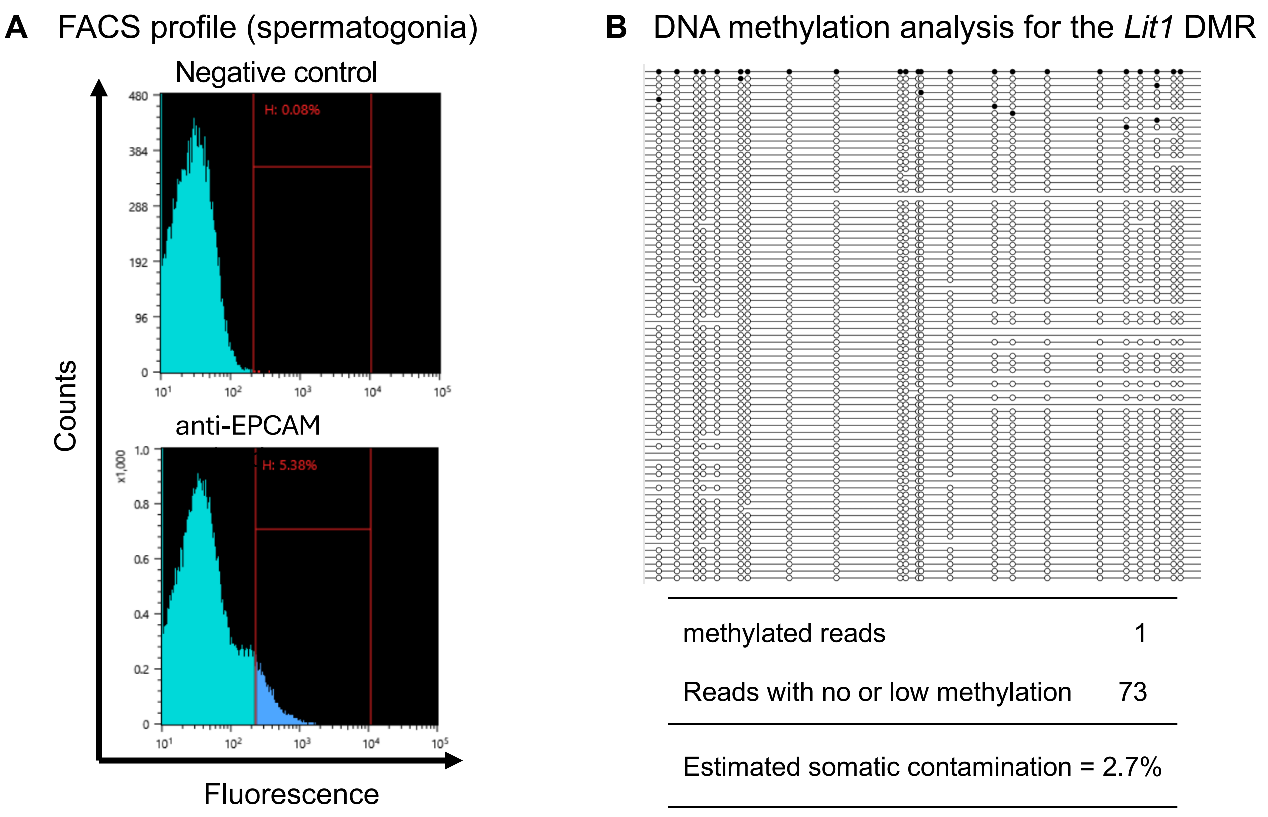

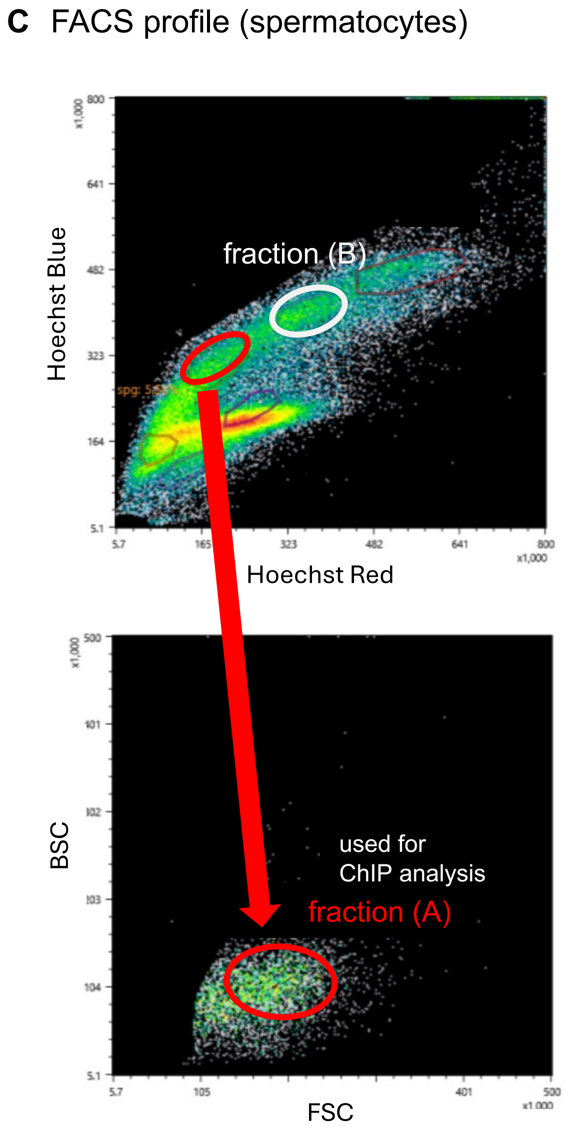

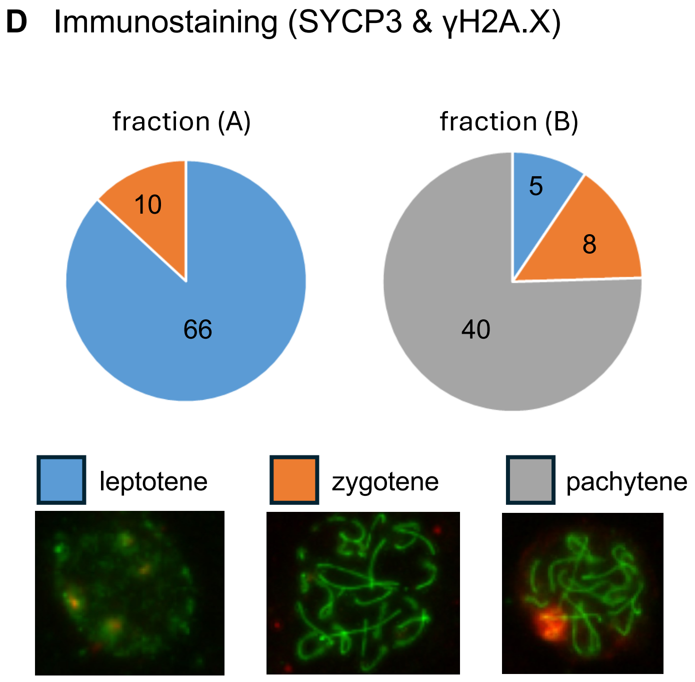

Legend is in the next page.

Figure S1. FACS profiles and cell-purity confirmation

(A) FACS profile for sorting EpCAM-positive spermatogonia. (B) Bisulfite-PCR results for the Lit1 DMR. The DNA was prepared from purified cells and bisulfite analysis was conducted as described previously (ref.15). Each raw represents a clone. Open circles, unmethylated CpGs. Close circles, methylated CpGs. (C) FACS profile for sorting L/Z spermatocytes. Fraction A was first gated based on Hoechst blue and red fluorescent intensities, then gated based on SSC and BSC, and used for ChIP-seq. Fraction B was analyzed as a control for pachytene-enriched fraction. (D) Statistics of respective meiotic stages in the sorted cell population. Typical Immunofluorescent images for leptotene, zygotene, and pachytene are shown below. Green, SYCP3. Red, γH2A.X.

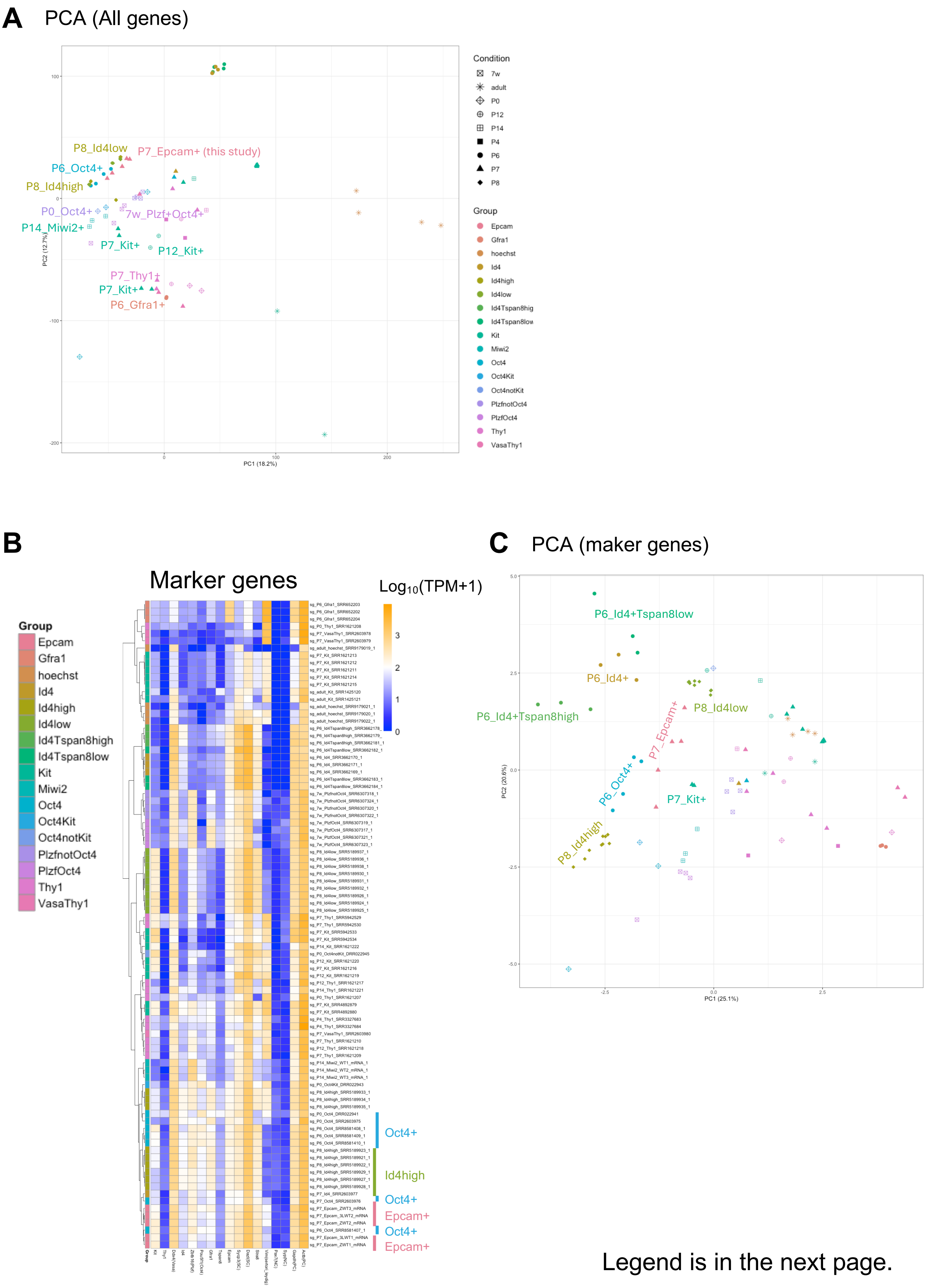

Legend is in the next page.

Figure S2. Comparison of gene expression profiles

(A) Principal component analysis of overall gene expression of various spermatogonia (wild-type). The sequence data of published results were obtained from Short Read Archive (SRA).

SRR3662169-36621701 (sg_P6_Id4), SRR3662178-3667181 (sg_P6_Id4Tspan8high), SRR3662182-2184 (sg_P6_Id4Tspan8low), SRR2603977 (sg_P7_Id4), SRR5189921-5189923, SRR5189927-5189929 and SRR5189933-5189935 (sg_P8_Id4high), SRR5189924-5189926, SRR51899230-5189932 and SRR5189936-5189938 (sg_P8_Id4low), SRR652202-652205 (sg_P6_Gfra1), SRR6307321 and SRR6307323 (sg_7w_PlzfOct4),), SRR6307322 and SRR6307324 (sg_7w_PlzfnotOct4), SRR2603975 and DRR022941 (sg_P0_Oct4), DRR022943 (sg_P0_Oct4Kit), DRR022945 (sg_P0_Oct4notKit), SRR8581407-8581409 (sg_P6_Oct4), SRR2603976 (sg_P7_Oct4), SRR2603978-2603980 (sg_P7_VasaThy1), SRR1621207 and SRR1621208 (sg_P0_Thy1), SRR3327683 and SRR3327684 (sg_P4_Thy1), SRR1621209, SRR1621210, SRR5942529and SRR5942530 (sg_P7_Thy1), SRR1621217 and SRR1621218 (sg_P12_Thy1), SRR1621211-1621216 and SRR4892879-4892880 (sg_P7_Kit), SRR1621219 and SRR1621220 (Sg_P12_Kit), SRR1621222 (Sg_P14_Kit).

The mRNA-seq data were mapped on to the mouse genome, and TPM values were calculated by stringtie. The PCA plot was created using Z-scaled TPM values of all genes.

(B) Heatmap representation of expression levels of marker genes in various spermatogonia.

(C) Principal component analysis using only marker genes (Kit, Thy1, Ddx4, Id4, Zbtb16, Pou5f1/Oct4, Gfra1, Tspan8, Epcam, Sycp3,DazL, Stra8, Vim, Pax7, Syp, Gapdh, and Actb) with Z scalling.

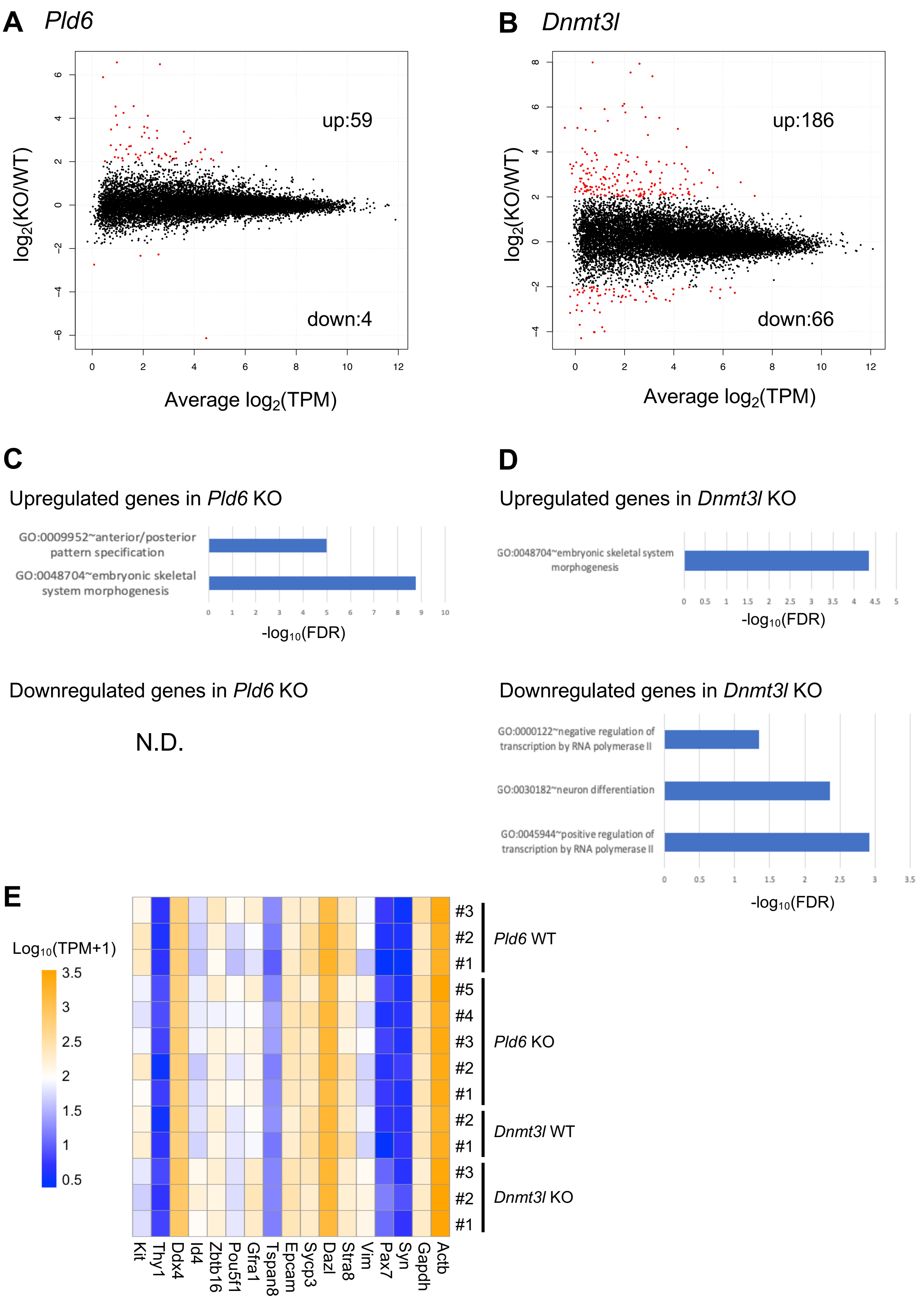

Figure S3. Gene expression changes in the KO mutants

(A) MA plot for gene expression between WT and *Pld6* KO spermatogonia. Significantly changed genes (>4 fold, q <0.05) were highlighted in red. (B) MA plot for gene expression between WT and *Dnmt3l* KO spermatogonia. Significantly changed genes (>4 fold, q <0.05) were highlighted in red. (C) Gene ontologies enriched in upregulated and downregulated genes in *Pld6* KO spermatogonia. N.D., none was detected. (D) Gene ontologies enriched in upregulated and downregulated genes in *Dnmt3l* KO spermatogonia. (E) Heatmap representation of expression levels of marker genes in wild-type and mutant spermatogonia.

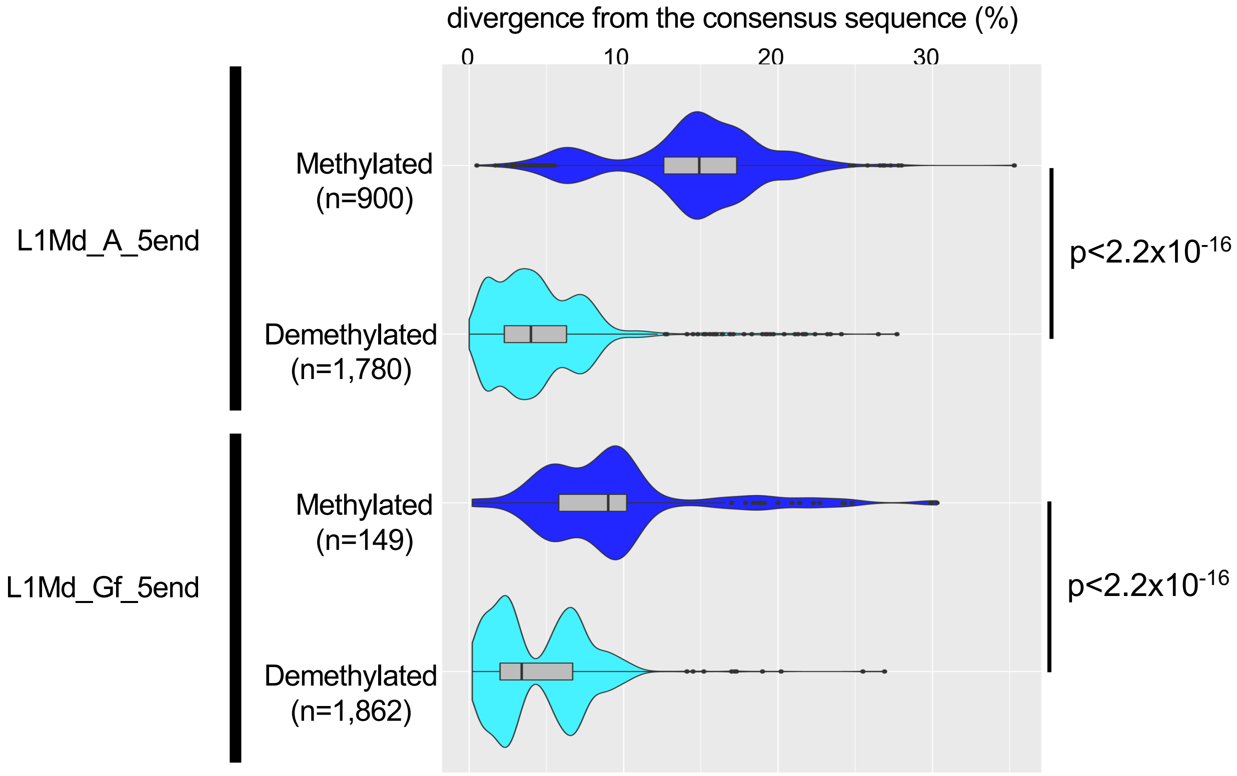

Figure S4. Nucleotide divergence of Pld6-independent and –dependent L1 copies

Violin plots for nucleotide divergence of the L1 subfamilies categorized by DNA methylation levels in *Pld6* KO spermatogonia. Methylated, ≧0.8 in WT and ≧0.8 in *Pld6* KO; demethylated, ≧0.8 in WT and ≦0.2 in *Pld6* KO. p-values (by U test) are indicated on the right.

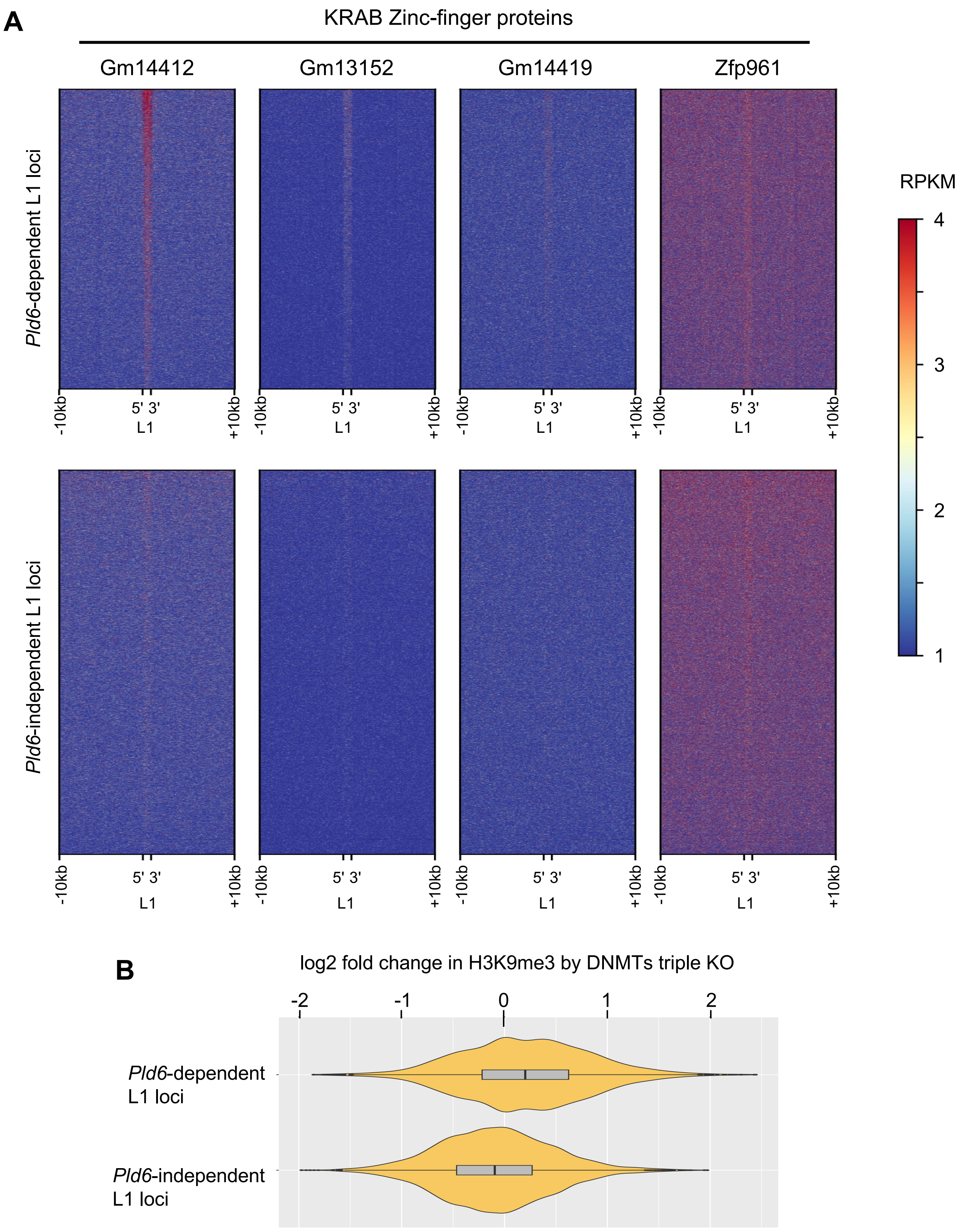

Figure S5. Binding of KRAB-ZFPs and changes of H3K9me3 at L1 loci in mouse ESCs

(A) Heatmap representation of ChIP-seq data of the four KRAB-ZFPs around the 5' L1 regions of which DNA methylation are *Pld6*-dependent (top) and *Pld6*-independent (bottom). Bigwig files were downloaded from GEO (GSE115291), and heatmaps were generated using DeepTools. (B) Violin plots for fold changes in H3K9me3 in *Dnmt1/Dnmt3a/Dnmt3b* triple KO ESCs. ChIP-seq data were downloaded from GEO (GSE29413).
